# Unique cellular immune signatures of multisystem inflammatory syndrome in children

**DOI:** 10.1371/journal.ppat.1010915

**Published:** 2022-11-02

**Authors:** Anuradha Rajamanickam, Pavan Kumar Nathella, Aishwarya Venkataraman, Poovazhagi Varadarjan, Srinithi Kannan, Arul Nancy Pandiarajan, Rachel Mariam Renji, Elayarani Elavarasan, Akshith Thimmaiah, Kandasamy Sasidaran, Nedunchelian Krishnamoorthy, Suresh Natarajan, Ganesh Ramaswamy, Balasubramanian Sundaram, Sulochana Putlibai, Syed Hissar, Elilarasi Selladurai, K. Ranganathan Uma Devi, Thomas B. Nutman, Subash Babu

**Affiliations:** 1 National Institutes of Health-National Institute for Research in Tuberculosis-International Center for Excellence in Research, Chennai, India; 2 ICMR-National Institute for Research in Tuberculosis, Chennai, India; 3 Institute of Child Health and Hospital for Children, Chennai, India; 4 Dr Mehta’s Children’s Hospital, Chennai, India; 5 Rainbow Children’s Hospital, Chennai, India; 6 Kanchi Kamakoti CHILDS Trust Hospital, Chennai, India; 7 Laboratory of Parasitic Diseases, National Institute of Allergy and Infectious Diseases, National Institutes of Health, Bethesda, Maryland, United States of America; Emory University, UNITED STATES

## Abstract

The clinical presentation of MIS-C overlaps with other infectious/non-infectious diseases such as acute COVID-19, Kawasaki disease, acute dengue, enteric fever, and systemic lupus erythematosus. We examined the *ex-vivo* cellular parameters with the aim of distinguishing MIS-C from other syndromes with overlapping clinical presentations. MIS-C children differed from children with non-MIS-C conditions by having increased numbers of naïve CD8^+^ T cells, naïve, immature and atypical memory B cells and diminished numbers of transitional memory, stem cell memory, central and effector memory CD4^+^ and CD8^+^ T cells, classical, activated memory B and plasma cells and monocyte (intermediate and non-classical) and dendritic cell (plasmacytoid and myeloid) subsets. All of the above alterations were significantly reversed at 6–9 months post-recovery in MIS-C. Thus, MIS-C is characterized by a distinct cellular signature that distinguishes it from other syndromes with overlapping clinical presentations.

**Trial Registration:** ClinicalTrials.gov clinicaltrial.gov. No: NCT04844242.

## Author summary

Multisystem inflammatory syndrome in children (MIS-C) is a severe disease that happens several weeks after severe acute respiratory syndrome coronavirus-2 (SARS-CoV-2) infection. We performed an in-depth analysis of immune cell subsets and demonstrated that children with MIS-C had a distinctive cellular signature that differentiates it from other overlapping clinical presentations. T, B and myeloid cellular compartments were altered in MIS-C children. Post-recovery (6–9 months post-treatment), most of these alterations were significantly reversed and returned to normal levels. Thus, MIS-C varies from acute COVID-19, other infectious, non-infectious diseases and healthy pediatric controls by exhibiting a unique cellular signature.

## Introduction

Children of all ages are at risk of SARS-CoV-2 infection [1], although the disease manifestation is mostly mild in the vast majority of infected children [2]. However, a small proportion of children may present with MIS-C, which develops 3 to 6 weeks following exposure to SARS-CoV-2. The clinical phenotype of MIS-C is wide-ranging and overlaps with that of Kawasaki disease (KD), MAS (Macrophage activation syndrome) and toxic shock syndrome (TSS). Some features of MIS-C are also similar to infectious diseases such as acute dengue, scrub typhus and enteric fever and non-infectious diseases such as diabetic ketoacidosis, juvenile idiopathic arthritis and Guillain-Barre syndrome. However, MIS-C [3,4] has a distinct set of immunological features that is different from adult COVID-19 or other diseases [5].

Flow cytometric immunophenotyping of MIS-C has shown lymphopenia with decreased absolute numbers of CD4^+^ and CD8^+^ cells and diminished expression of HLA-DR on monocytes and dendritic cells [5]. Similarly, decreased absolute numbers of CD16^+^ monocytes, and plasmacytoid dendritic cells have been reported in MIS-C children [6] with other populations of cells (e.g., naïve and effector B and T cells) remaining unperturbed. To identify distinctive parameters in MIS-C and in acute pediatric COVID-19, we used multiparameter immune profiling in MIS-C and in acute COVID-19 and compared these profiles to those of other infectious or non-infectious diseases with overlapping clinical features and to healthy pediatric controls.

## Results

### Characteristics of the study population

A total of 98 children with a median age of 6.5 years (range: 1 – 15yr) were included whose demographic, clinical and laboratory details are shown in Table 1. This includes MIS-C (n = 21), Acute COVID-19 (S2 Table; n = 23), other infectious diseases (S3 Table; n = 19; Dengue fever = 3, Enteric fever = 3, Scrub typhus = 5, other etiology = 8), non-infectious diseases [S4 Table; n = 21; Diabetic Ketoacidosis = 6, Kawasaki’s Disease = 3, Juvenile Idiopathic Arthritis (JIA) = 1, Guillain-Barré syndrome (GBS) = 3, Systemic Lupus Erythematosus (SLE) = 4, chronic renal failure = 3] and healthy controls (n = 14). C-Reactive Protein (CRP) levels were significantly elevated in children with MIS-C when compared with those with acute COVID-19, other infectious and non-infectious diseases and healthy control children (Table 1). We also analyzed the *ex vivo* phenotype and hematological parameters in a subset of MIS-C children post-recovery (following 6–9 months of recovery) (Fig 4).

**Table 1:** Characteristics of the study population.

	Acute COVID-19 n = 23	MIS-C n = 21	Other infectious diseases n = 19*	Non-infective diseases n = 21^#^
**Age median (years, IQR)**	6 (1–17 yr)	5.9 y (1 – 12y)	4.5 y (1–12 y)	7 y (2 – 12y)
**Male n (%)**	9 (39%)	11 (52%)	12 (63%)	10 (48%)
**RT-PCR positive n (%)**	23 (100%)	0	0	0
**Serology IgG positive n (%)**	0	21 (100%)	0	5 (24%)
**Underlying conditions n (%)**	4 (17%)^**£**^	3 (14%)^a^	1(23%) ^c^	21 (100)^**#**^
**Symptoms n (%)**				
*Fever*	19 (83%)	21 (100%)	10 (53%)	10 (48%)
*Gastrointestinal*	12 (52%)	15 (72%)	8 (42%)	14 (66%)
*Respiratory*	5 (22%)	3 (14%)	5 (26%)	6 (29%)
*Mucocutaneous*	0	15 (72%)	2 (10%)	3 (14%)
*Asymptomatic*	2 (8%)	0	0	0
**Cardiovascular symptoms/signs**				
*Hypotension*	1	11 (52%)	6 (32%)	3 (14%)
*Coronary artery dilatation*	0	3	0	0
*Mycocardial dysfunction*	0	11 (52%)	6 (32%)	3 (14%)
**Laboratory parameters**	**n = 14**	**n = 21**	**n = 19**	**n = 21**
***CRP (< 3 mg/L) median (IQR)***	9.4 (<3–48.0)	32.9 (3.5–196.7)	21.3 (<3–78)	NA
***Sodium (135 – 145mmol/l) median (IQR)***	138 (136–145)	131 (124–139)	134 (131–148)	137 (133–143)
***Ferritin (ng/mL) (7 to 140) median (IQR)***	NA	1348 (306–5377)	440 (13–3752)	614 (42–7200)
**Median duration of stay (days)**	4 (1–9)	6 (3–18)	5 (1–20)	11 (3–21)
***PICU admission***	1 (4%)	11 (52%)	8 (42%)	12 (57%)
**Treatment n (%)**				
***IVIG***	0	7 (33%)	0	3 (14%)
***Steroids***	1 (4%)	15 (72%)	3 (16%)	5 (24%)
***Antibiotics***	3 (13%)	21 (100%)	12 (63%)	21 (100%)
***Tocilizumab (8mg/kg)***	0	1 (5%)	0	0
**Respiratory support n (%)**				
***Mechanical Ventilation***	0	1 (5%)	5 (26%)	6 (29%)
***HHFNC***	1 (4%)	3 (14%)	2 (10%)	3 (145)
***Oxygen***	0	2 (9%)	1 (5%)	0
**Cardiovascular support n (%)**				
***Inotropes***	0	11 (52%)	6 (32%)	3 (14%)
***Fluid Bolus***	1 (4%)	15 (72%)	2 (10%)	4 (19%)

* Nephrotic syndrome, Dilated Cardiomyopathy, Seizure disorder, ADHD

^£^Details Diagnosis: Dengue fever, Scrub typhus, Typhoid, Acinetobacter sepsis, Urinary tract infection; Clinical symptom: No microorganism isolated Neurodevelopmental delay

^**#**^ Underlying Diagnosis: Diabetic ketoacidosis, Kawasaki’s disease, Juvenile idiopathic arthritis, Guillain-Barre syndrome, Systemic lupus erythematosus, Chronic renal failure. Clinical Symptoms: Fever, Respiratory, Gastrointestinal, Mucocutaneous, Neuromuscular (Headache, abnormal gait etc), Renal, Hypothyroidism.PICU: paediatric intensive care unit; HHFNC: high flow nasal cannula oxygen; IVIG: intravenous immunoglobulin, CRP: C—reactive protein.

^a^ nephrotic syndrome, dilated cardiomyopathy, Asthma

^c^ Neurodevelopmental delay

### CD4^+^ and CD8^+^ T cell subsets are altered in MIS-C

**Fig 1:**
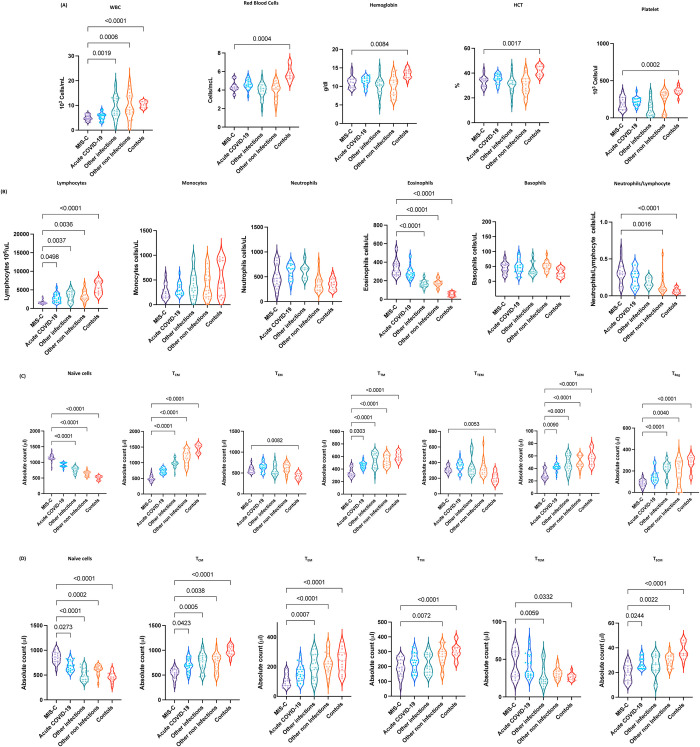
CD4^+^ and CD8^+^ T cell subsets are altered in MIS-C. (A) Analysis of WBC, RBC, Hb, HCT and platelets (B) Analysis of absolute count of WBC, lymphocytes, monocytes, neutrophils, eosinophils, basophils and neutrophils/lymphocyte ratio were shown for children with MIS-C (n = 21), acute COVID-19 (n = 23), other infectious (n = 23), other non-infectious diseases (n = 21) and controls (n = 14). (C) Absolute numbers of CD4^+^ T cell subsets in MIS-C (n = 21), acute COVID-19 (n = 23), other infectious (n = 23), other non-infectious diseases (n = 21) and controls (n = 14). The data are represented as scatter violin plots with each circle representing a single individual. p values were calculated using the Kruskal-Wallis test with Dunn’s post-hoc for multiple comparisons. (D) Absolute numbers of CD8^+^ T cell subsets in MIS-C (n = 21), acute COVID-19 (n = 23), other infectious (n = 23), other non-infectious diseases (n = 21) and controls (n = 14). The data are represented as scatter violin plots with each circle representing a single individual. p values were calculated using the Kruskal-Wallis test with Dunn’s post-hoc for multiple comparisons.

To determine the effect of MIS-C on laboratory parameters, we assessed the percentage and absolute numbers of laboratory parameters in MIS-C children and compared them with acute COVID-19, other infectious and non-infectious diseases and healthy control children. As shown in Fig 1A, children with MIS-C had significantly decreased WBC counts, RBC, hemoglobin, HCT and platelets counts when compared with other infections, other non-infections and control children. As shown in Fig 1B, absolute numbers of lymphocytes were markedly decreased in children with MIS-C when compared with acute COVID-19 children. MIS-C children also exhibited decreased absolute numbers of lymphocytes when compared with other infections, other non-infections and control children. MIS-C children had significantly decreased absolute numbers of monocytes when compared with other infections, other non-infections and control children. In contrast, MIS-C children had significantly increased numbers of eosinophils when compared with other infections, other non-infections and control children. Also, MIS-C children had significantly elevated neutrophil/lymphocyte ratio when compared with other non-infections and controls.

To determine the effect of MIS-C on memory CD4^+^ T cell subsets, we assessed the absolute numbers of CD4^+^ T cell subsets by flow cytometry in MIS-C children and compared them with acute COVID-19, other infectious and non-infectious diseases and healthy control children. A representative plot showing the gating strategy for CD4^+^ T cell subsets is shown in S1 Fig. As shown in Fig 1C, within the CD4^+^ T cell compartment, children with MIS-C exhibited decreased absolute numbers of transitional memory cells, stem cell memory and regulatory CD4^+^ T cells when compared with acute COVID-19 children. MIS-C children also exhibited significantly increased absolute numbers of naïve CD4^+^ T cells compared to children with other infectious and non-infectious diseases. In contrast, MIS-C children exhibited decreased absolute numbers of central memory, transitional memory, stem cell memory and regulatory CD4^+^ T cells in comparison to children with other infectious and non-infectious diseases. MIS-C children also exhibited increased absolute numbers of naïve cells, effector memory cells and terminal effector memory CD4^+^ T cells in comparison to the control group of children. In contrast, the absolute numbers of central memory cells, transitional memory cells, stem cell memory and regulatory CD4^+^ T cells were significantly increased in children with MIS-C in comparison to the control group of children. Thus, MIS-C children are associated with altered absolute numbers of CD4^+^ T cell subsets.

To determine the impact of MIS-C on the memory CD8^+^ T cell compartment, we assessed the absolute numbers of memory CD8^+^ T cell subsets by flow cytometry in MIS-C children and compared them with acute COVID-19, other infectious and non-infectious diseases and healthy control children. A representative flow cytometry plot showing the gating strategy for CD8^+^ T cell subsets is shown in S1B Fig. As shown in Fig 1D, within the CD8^+^ T cell compartment, children with MIS-C exhibited increased absolute numbers of naïve cells in comparison to children with acute COVID-19. In contrast, absolute numbers of central memory and stem cell memory CD8^+^ T cells were significantly increased in MIS-C children in comparison to children with acute COVID-19. MIS-C children also exhibited significantly increased absolute numbers of naïve cells and terminal effector memory CD8^+^ T cells in comparison to children with other infectious diseases. In contrast, MIS-C children exhibited absolute decreased numbers of central memory, effector memory and terminal effector memory CD8^+^ T cells in comparison to children with other infectious diseases. Also, absolute numbers of naive CD8^+^ T cells were significantly increased in MIS-C children in comparison to children with other non-infectious diseases. In contrast, the absolute numbers of central memory, effector memory, transitional memory and stem cell memory CD8^+^ T cells were significantly decreased in MIS-C children in comparison to children with other non-infectious diseases. MIS-C children also exhibited increased absolute numbers of naïve cells and terminal effector memory CD8^+^ T cells in comparison with the control group of children. In contrast, the absolute numbers of central memory cells, effector memory, transitional memory cells and stem cell memory CD8^+^ T cells were significantly decreased in children with MIS-C in comparison with the control group of children. Our results show that MIS-C children are associated with altered absolute numbers of CD8^+^ T cell subsets.

### B cell subsets are altered in MIS-C

**Fig 2:**
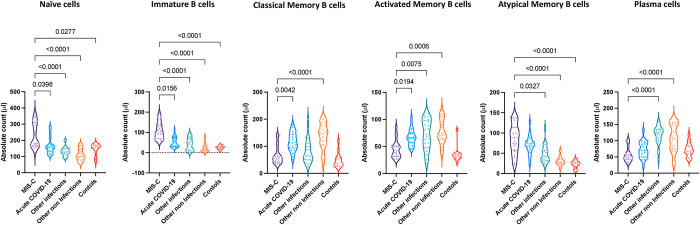
B cell subsets are altered in MIS-C. Absolute numbers of B cell subsets in MIS-C (n = 21), acute COVID-19 (n = 23), other infectious (n = 23), other non-infectious diseases (n = 21) and controls (n = 14). The data are represented as scatter violin plots with each circle representing a single individual. p values were calculated using the Kruskal-Wallis test with Dunn’s post-hoc for multiple comparisons.

Next, we examined the absolute numbers of B cell subsets by flow cytometry in MIS-C children and compared them with acute COVID-19, other infectious and non-infectious diseases and healthy control children. A representative flow cytometry plot showing the gating strategy for B cell subsets is shown in S2 Fig. As shown in Fig 2, within the B cell compartment, children with MIS-C exhibited increased absolute numbers of naïve B cells, immature B cells and atypical memory B cells in comparison to children with acute COVID-19. However, we also found that children with MIS-C had significantly decreased absolute numbers of classical memory, activated memory B cells and plasma cells in comparison to children with acute COVID-19. Children with MIS-C exhibited increased absolute numbers of naïve, immature and atypical memory B cells in comparison to children with other infections, other non-infections and controls. In contrast, the absolute numbers of activated memory B cells and plasma cells were decreased in children with MIS-C in comparison to children with other infections. In addition, the absolute numbers of classical memory and activated memory B cells were decreased in children with MIS-C in comparison to children with other non-infectious diseases. Our results show that MIS-C children are associated with altered absolute numbers of B cell subsets.

### Altered numbers of DC and monocyte subsets are associated with MIS-C

**Fig 3:**
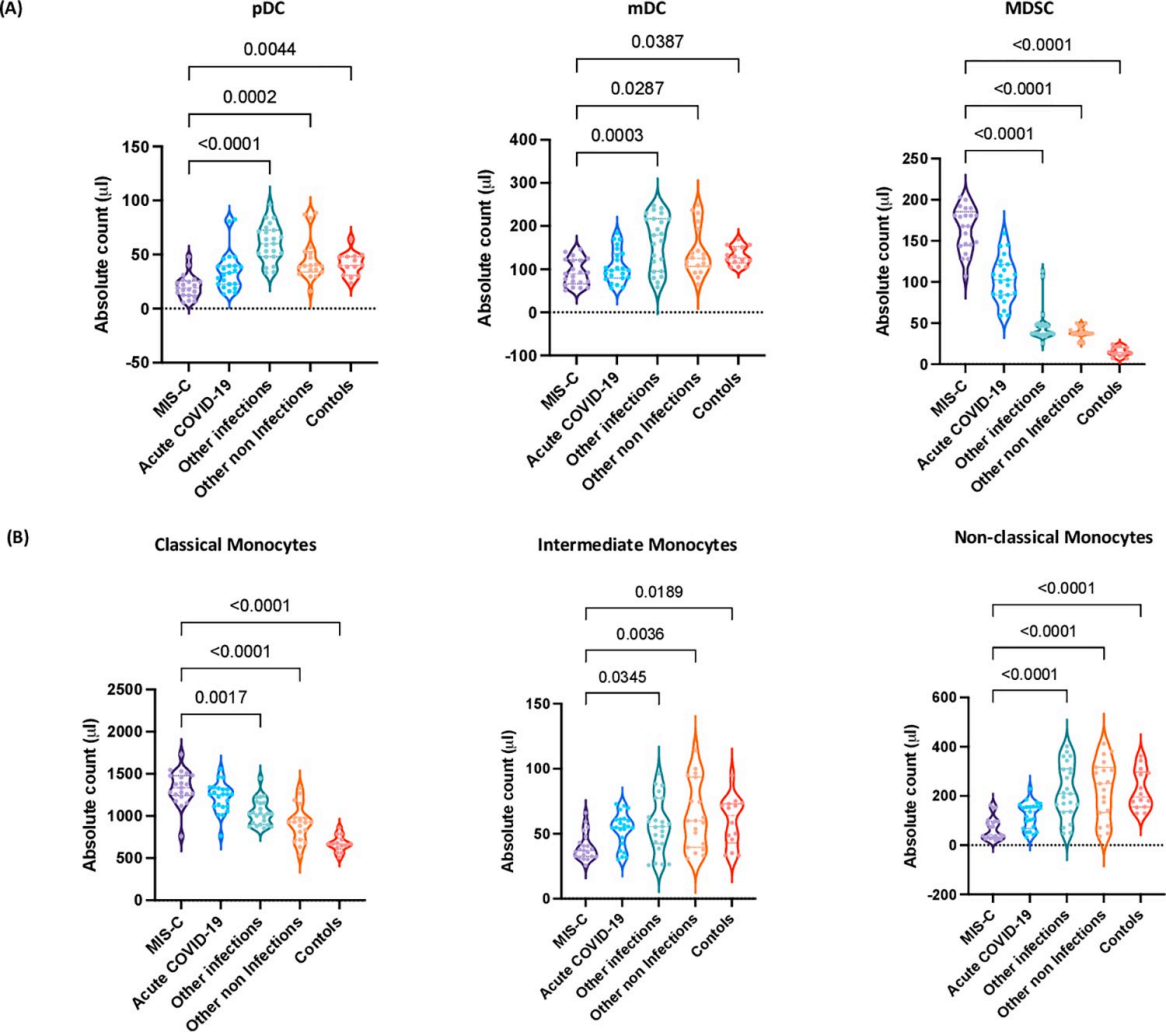
Altered numbers of DC and monocyte subsets are associated with MIS-C. **Title.** (A) Absolute numbers of DC subsets in MIS-C (n = 21), acute COVID-19 (n = 23), other infectious (n = 23), other non-infectious diseases (n = 21) and controls (n = 14). The data are represented as scatter violin plots with each circle representing a single individual. p values were calculated using the Kruskal-Wallis test with Dunn’s post-hoc for multiple comparisons. (B) Absolute numbers of monocyte subsets in MIS-C (n = 21), acute COVID-19 (n = 23), other infectious (n = 23), other non-infectious diseases (n = 21) and controls (n = 14). The data are represented as scatter violin plots with each circle representing a single individual. p values were calculated using the Kruskal-Wallis test with Dunn’s post-hoc for multiple comparisons.

Next, we examined the absolute numbers of DC and monocyte subsets by flow cytometry in children with MIS-C and compared them with acute COVID-19, infectious diseases, other non-infectious diseases and control children. A representative flow cytometry plot showing the gating strategy for DC subsets is shown in S3A and S3B Fig. As shown in Fig 3A, within the DC compartment, MIS-C children exhibited decreased absolute numbers of pDC and mDC in comparison to children with other infections, other non-infections and controls. In contrast, MIS-C children exhibited significantly increased absolute numbers of MDSC in comparison to children with other infections, other non-infections and controls. Finally, we examined the absolute numbers of classical, intermediate and non-classical monocytes by flow cytometry in children with MIS-C and compared them with acute COVID-19, other infectious diseases, non-infectious diseases and control children. A representative flow cytometry plot showing the gating strategy for monocyte subsets is shown in S3C Fig. As shown in Fig 3B, within the monocyte subsets, MIS-C children exhibited increased absolute numbers of classical monocytes in comparison to children with other infections, other non-infections and controls. In contrast, the absolute numbers of intermediate and non-classical monocytes were significantly decreased in comparison to children with other infections, other non-infections and controls. Thus, MIS-C children are associated with altered absolute numbers of myeloid cell subsets.

### T cell, B cell, DC and monocyte subsets were altered 6–9 months post recovery in MIS-C

**Fig 4:**
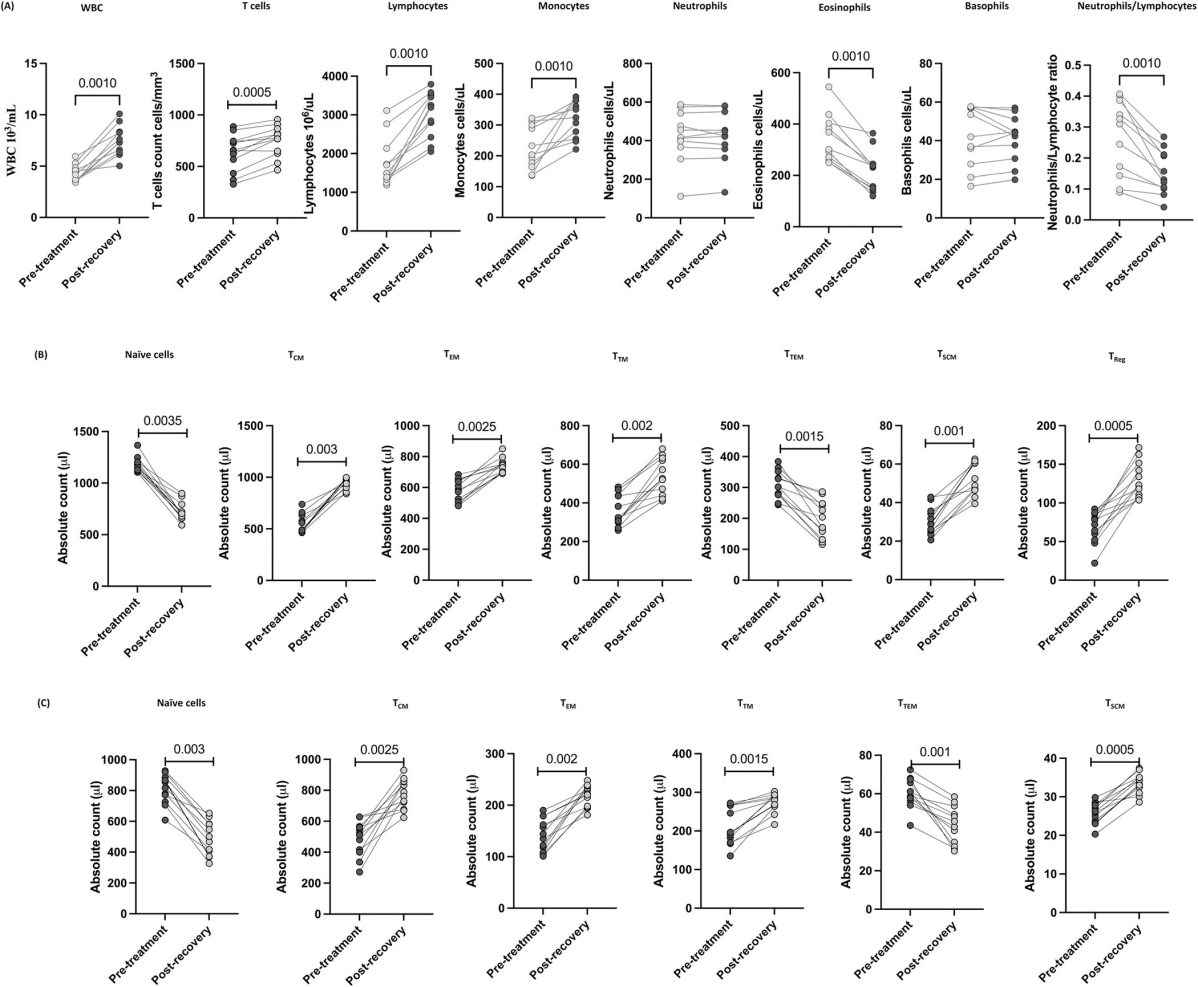
T cell subsets were altered post-recovery in MIS-C. (A) Absolute numbers of CD4^+^ T cell subsets in MIS-C children (n = 12) at pre-treatment and post-recovery were determined. (B) Absolute numbers of CD8^+^ T cell subsets in MIS-C children (n = 12) at pre-treatment and post-recovery were determined. p values were calculated using the Wilcoxon matched pair test.

Next, we wanted to assess the impact of treatment and 6–9 months of recovery on children with MIS-C. To this end, we examined the absolute numbers of T cells, B cells, DC and monocyte subsets at 6–9 months post-recovery and compared them with pre-treatment numbers. As shown in Fig 4A and 4B, within the T cell compartment, CD4^+^ and CD8^+^ naive and terminal effector T cell subsets were significantly decreased compared to pre-treatment levels. In contrast, the CD4^+^ and CD8^+^ T cell subsets such as central memory, effector memory, and transitional and stem cell memory were significantly increased compared to pre-treatment. Similarly, regulatory T cells were also significantly increased compared to pre-treatment levels.

**Fig 5:**
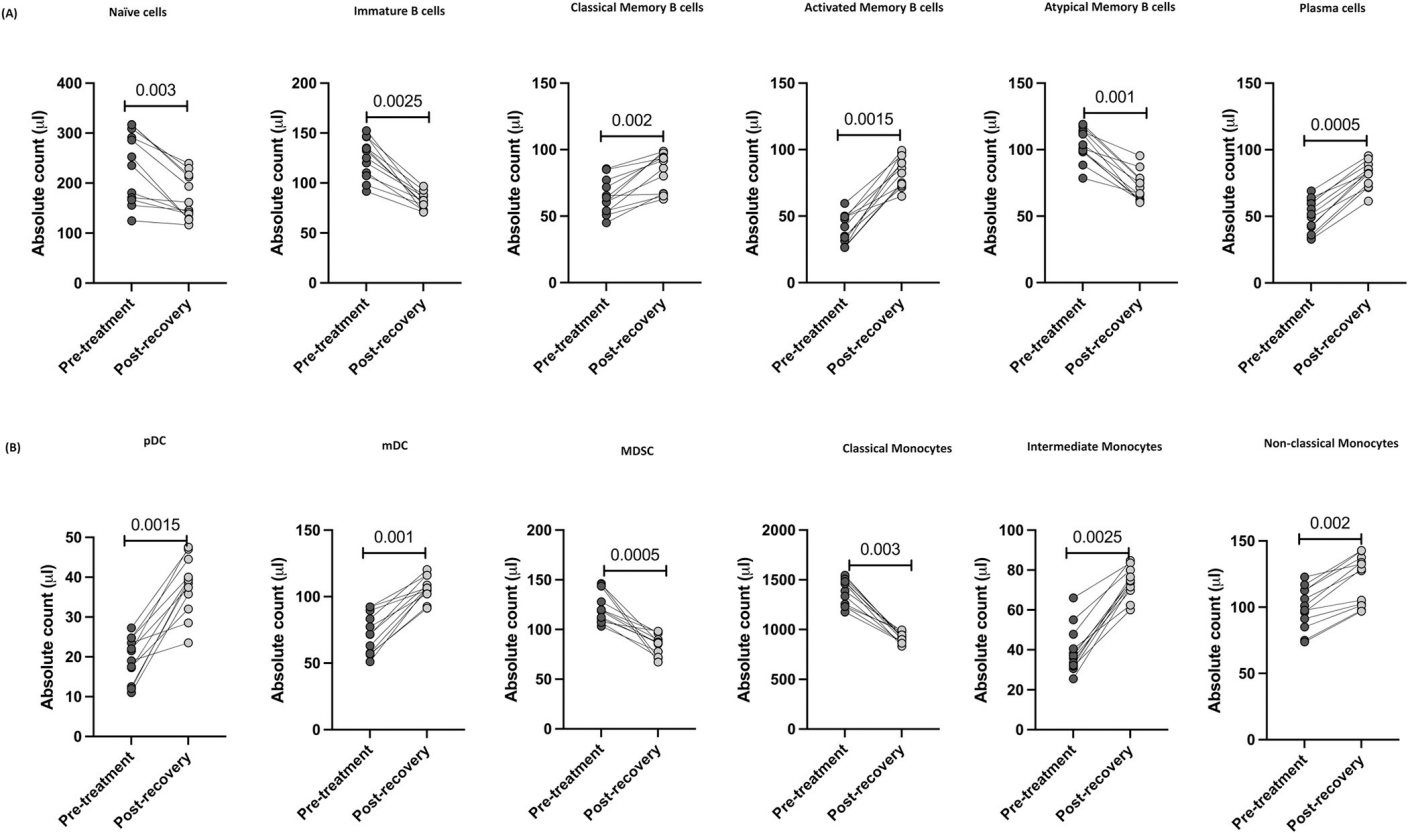
B cell, DC and monocyte subsets were altered post-recovery in MIS-C. (A) Absolute numbers of B cell subsets in MIS-C children (n = 12) at pre-treatment and post-recovery were determined. (B) Absolute numbers of DC and Monocyte subsets in MIS-C children (n = 12) at pre-treatment and post-recovery were determined. p values were calculated using the Wilcoxon matched pair test.

Subsequently, we determined the absolute numbers of B cell subsets 6–9 months post-recovery. As shown in Fig 5A, within the B cell compartment, naïve B cells, immature B cells and atypical memory B cells were significantly decreased compared to pre-treatment levels. In contrast, classical memory B cells, activated memory B cells and plasma cells were significantly increased compared to pre-treatment levels. As shown in Fig 5B, pDC, mDC, intermediate and non-classical monocytes were significantly increased whereas, the numbers of MDSC and classical monocytes were significantly decreased compared to pre-treatment levels. Thus, MIS-C children are associated with restoration and significant alteration of absolute numbers of T cells, B cells and myeloid cell subsets in children 6–9 months post-recovery.

Also, we wanted to compare the post-recovery levels in MIS-C with control children at baseline. As shown in S4 Fig., post-recovery levels of hematological and immunological parameters in MIS-C children were still significantly different from levels in control children.

### Hematological parameters and cellular subsets can strongly discriminate MIS-C from acute COVID-19 and other infections, other non-infections and control children

**Fig 6:**
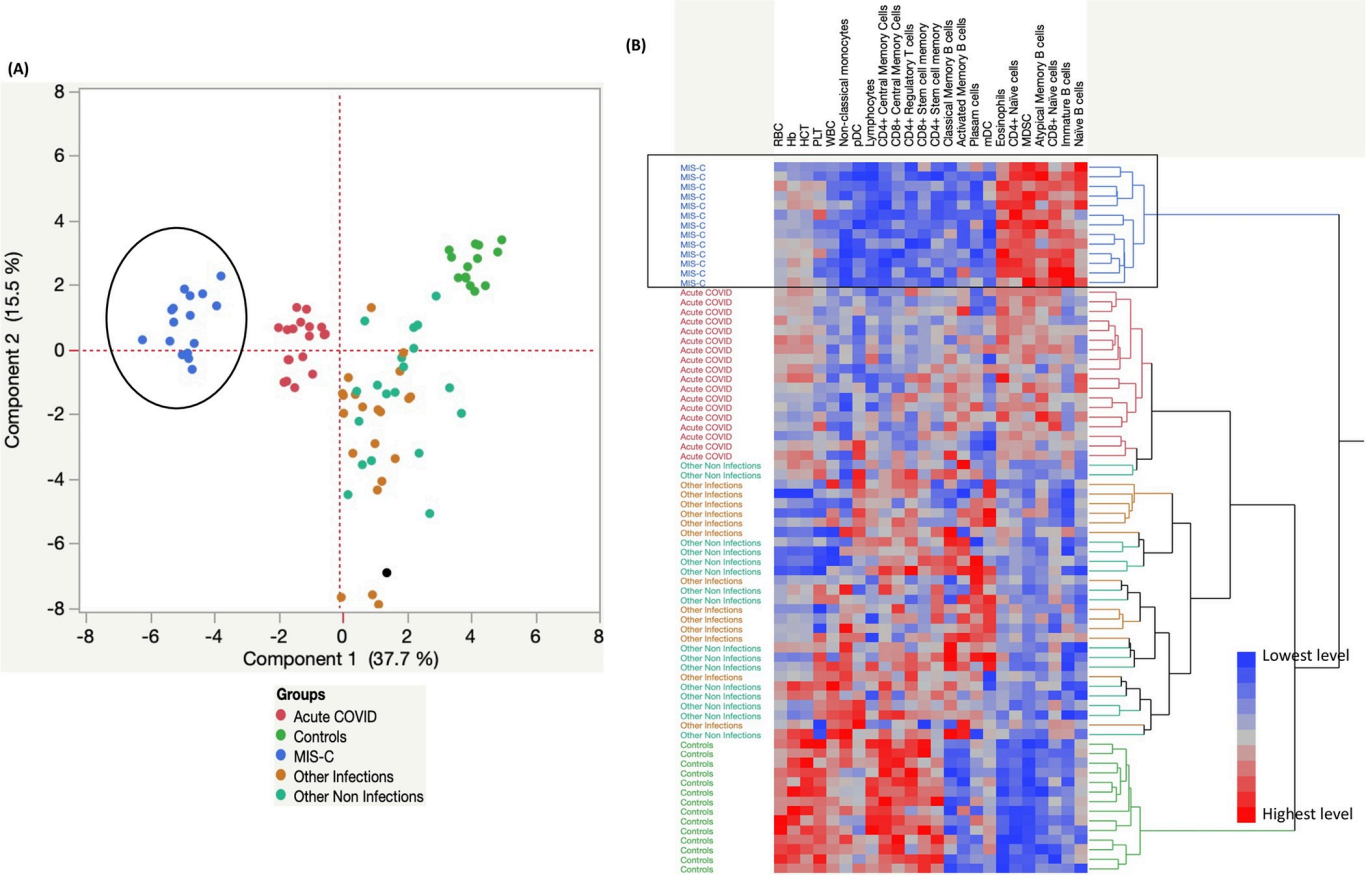
Hematology and Cellular subset markers can strongly discriminate MIS-C from acute COVID-19 and other infections, other non-infections and control children. **(A).** PCA (Principal component analysis) plot computing normalized cellular subsets after excluding those factors with commonalities as low as 0.5 we used hematology parameters like RBC, Hb, HCT, PLT, WBC, lymphocytes and eosinophils, cellular subsets absolute counts of CD4^+^ and CD8^+^ Naïve, central memory, stem cell memory, Regulatory T cells, Naïve B cells, Immature B cells, Classical B cells, Activated, Atypical memory B cells, plasma cells, pDC, mDC, MDSC and non-classical monocytes in a combination of five different experimental groups first MIS-C (Colored in blue) vs acute COVID-19 (Colored in red) vs other infections (Colored in brown) vs other non-infections (Colored in light green) and controls (Colored in dark green). The PCA shows the two principal components of variation, accounting for 15.5% (x-axis) and 37.7% (y-axis). **(B).** Hematology and cellular subsets are illustrated according to the score denoted in the color-scale bar. Associated horizontal dendrograms denote the patient’s clustering, standing out clusters containing MIS-C (enclosed in blue), acute COVID-19 children (enclosed in red), other infections (Colored in brown), and other non-infections (enclosed in green) or controls children (enclosed in light green). On the color scale, blue color indicates lower expression and red color indicates higher expression.

We performed PCA (principal component analysis) plot computing normalized hematology and cellular subsets after excluding those factors with commonalities as low as 0.5 and used the hematological parameters like RBC, Hb, HCT, PLT, WBC, lymphocytes, eosinophils and absolute counts of CD4^+^ and CD8^+^ Naïve, central memory, stem cell memory, Regulatory T cells, Naïve B cells, Immature B cells, Classical B cells, Activated, Atypical memory B cells, plasma cells, pDC, mDC, MDSC and non-classical monocyte subsets to determine the discriminatory ability of cellular subsets (Fig 6A) in distinguishing in children with MIS-C from acute COVID-19, other infections, other non-infections and control children. Further, we performed hierarchical clustering analysis, using data sets that included the absolute numbers of CD4^+^, CD8^+^ memory T cell subsets, B cell and myeloid cell subsets from all five groups of children. A heatmap and dendrogram for cellular subsets (Fig 6B) are depicted. As shown, the dendrogram generated using Ward’s supervised clustering method and index is able to discriminate the spectrum of MIS-C, acute COVID-19, other infections, other non-infections and controls as clearly defined clusters.

## Discussion

We have previously described the immune profile of different presentations of SARS-CoV-2 infection (MIS-C, acute COVID-19 and seropositive children) [7]. Here, we have described and compared the immune cell subsets in MIS-C and compared them to those with acute COVID-19 and with other infectious and non-infectious diseases with overlapping clinical presentations. This comparison and data are pivotal in low- and middle-income countries (LMIC) and tropical countries where diseases such as dengue, scrub typhus and enteric fever are endemic. While immunological variation involving alterations in the T cell compartment during deep immune profiling of children with MIS-C and COVID-19 have been previously described, [5,6,8], we report a high dimensional whole blood flow cytometric immune profile from an LMIC for the first time, to our knowledge. Decreased lymphocyte counts are a consistent finding and are associated with worse outcomes in the adult COVID-19 [9,10]. Similarly, previous studies on MIS-C revealed that children with MIS-C present with lymphopenia and neutrophilia [11,12]. Consistent with this, we observed lower levels of leukocytes, lymphocytes, and slightly elevated neutrophils along with low hemoglobin, and a lower number of monocytes and eosinophils in children with MIS-C (Table 1). Earlier studies showed that the levels of lymphocytes, eosinophils, and platelets are normalized post-treatment and might perform as prognosticators for the recovery [13,14]. Similar to previous studies, we also found that the levels of hemoglobin, hematocrit, platelet, lymphocytes and monocytes were increased 6–9 months post-recovery. Four reports describing T cell responses in pediatric COVID-19 revealed either similar T cell counts to healthy pediatric controls or higher counts in comparisons with adult patients [15–18]. Our study also innovates by analyzing the *ex-vivo* immune cellular subset profile in a subset of children with MIS-C and demonstrates that 6–9 months post-recovery, MIS-C children exhibit significant alterations in cellular subset numbers.

Previous studies have described reductions in the helper (CD4^+^), cytotoxic (CD8^+^), total T cell counts [5] and an increase in naïve cells, central, effector memory T cells along with a reduction in regulatory T cells in children with MIS-C [3]. Likewise, increased absolute numbers of circulating memory T cells in children with acute COVID-19 have been reported [6]. MIS-C could be associated with perturbed innate and adaptive cell absolute numbers, comprising a deficiency of T cells and augmented memory T cell activation associated with healthy pediatric controls [5,6,19]. Among the non-infectious diseases, in a pediatric study, surface marker expression was related to a stimulated T-cell phenotype that was elevated in asymptomatic viremic individuals in comparison to clinical dengue individuals [20,21]. In the pathogenesis of lupus, innate immune cells such as dendritic cells (DCs) as well as adaptive immune cells including B and T cells are involved [22]. Consistent with these reports, our data also revealed that MIS-C children exhibited enhanced absolute numbers of naïve cells, central, effector memory T cells and diminished absolute numbers of effector T cells, stem cell memory T cells and regulatory T cells. We also found that alterations in the T cell subsets encompass both the CD4^+^ and CD8^+^ T cell compartments and are likely the result of enhanced apoptosis of T cells and/or migration of T cells into the tissue compartments from the periphery. We also identified significant differences in the transitional memory, stem cell memory, central and effector memory CD4^+^ and CD8^+^ T cell subsets between MIS-C and acute COVID-19 children. Recent studies indicate that the Treg count in peripheral blood is diminished in adults with COVID-19, especially in severe cases [9,23,24]. The CD4^+^ Treg absolute numbers in pediatric COVID-19 have been recently described [25] and our study also revealed similar findings of decreased absolute numbers of Treg. In addition, we observed that children with MIS-C and acute COVID-19 exhibited decreased absolute numbers of Tregs in comparison to children with other infections, non-infectious diseases and control groups. We also found that the CD4^+^ and CD8^+^ absolute numbers of naïve cells and terminal effector memory cells were significantly reduced post-recovery in comparison to pre-treatment values. In contrast, the absolute numbers of CD4^+^ and CD8^+^ central memory, effector memory, transitional memory and stem cell memory were significantly increased post- recovery compared to pre-treatment numbers. Recent data indicate a remarkable expansion of regulatory T cell counts in the convalescence phase of MIS-C and COVID-19 [5]. Consistent with this, we found that regulatory T cell numbers were increased 6–9 months post-recovery.

Low levels of total B cells, effector B cells and class-switched memory B cells [26] and elevated immature plasma B cells and skewed B cell responses [27] have been reported in MIS-C. MIS-C children from our study showed similar responses with enhanced absolute numbers of immature B cells along with diminished absolute numbers of activated memory B cells and plasma cells when compared with acute COVID-19. Similar to the previous reports by Consiglio et al and Weisberg et al, [19,28] we also found that the absolute numbers of naïve B cells, and atypical memory B cells were significantly increased in our group of MIS-C and acute COVID-19. MIS-C children exhibited increased numbers of naïve cells, immature B cells, and atypical memory B cells in comparison with other infections and other non- infections children. In contrast, classical memory, activated and plasma cells numbers were significantly reduced in MIS-C children in comparison to other infection and other non- infections children. Carter JM et al revealed that in MIS-C, during the acute phase the total number and effector B cells were reduced and class switch memory B cell numbers were increased in the resolution phase [5]. We observed that 6–9 months post-recovery, the numbers of naïve, immature and atypical memory B cells were reduced whereas the numbers of classical memory, activated memory and plasma cells were increased in comparison to pre-treatment numbers. Further studies are necessary to confirm and understand the mechanisms behind these alterations.

Recent studies have reported the distribution of monocytes and DC in MIS-C and acute COVID-19 in children highlighting a reduction in total monocyte and DC count [27] as well as decreased HLA-DR expression and increased non-classical monocytes [5,6,29]. In SLE and RA, more differentiated monocyte subsets play an essential role in the disease progression [30]. Our study adds to the existing evidence that MIS-C and acute COVID-19 children exhibit reduced DC subsets (pDC and mDC), intermediate and non-classical monocytes but show enhanced classical monocytes. MDSCs are innate immune cells and link innate and adaptive immunity [31]. Recent studies divulged that severe COVID-19 patients exhibited a dysregulated myeloid cell compartment, with augmented MDSC levels and activity associated with disease severity [31]. To our knowledge, this is the first study showing an expansion of MDSC in children with MIS-C in comparison to acute COVID-19, other infectious diseases, non-infectious diseases and control children. While T cells appear to be more activated in MIS-C, antigen-presenting cells like monocytes, DC and B cells have lower markers of activation, suggesting a possible deficiency in antigen presentation. In children with MIS-C discharged from the hospital, Syrimi et. al. revealed that the frequency of classical monocytes were decreased compared to their pre-treatment levels [32]. Likewise, we also found that the absolute numbers of MDSC and classical monocytes were significantly reduced. In contrast, the absolute numbers of pDC, mDC, intermediate and non-classical monocytes were significantly increased when compared to their pre-treatment numbers. The consequence and mechanisms sustaining these myeloid cell alterations in MIS-C need additional assessment.

To our knowledge, this is the first study to explore the difference in immunological responses between COVID-19, MIS-C, infectious (Dengue fever, Typhoid fever, Scrub typhus), and non-infectious diseases (DKA, SLE) in pediatric populations in LMIC. Our study examined the immune cellular and cytokine responses in a variety of infectious/ non-infectious conditions. These include conditions that typically mimic MIS-C such as KD, Dengue hemorrhagic shock and diabetic ketoacidosis, which make the clinical diagnosis of MIS-C a major challenge. Our study not only provides important knowledge about the pathophysiology of MIS-C but also illustrates a significant difference in the immune profile between MIS-C and acute COVID as well as other infectious/ non-infectious diseases that are common in LMICs like India.

In conclusion, we are able to provide unique immune signatures using *ex-vivo* profiling of immune cell subsets (T cell, B cell, DC and monocyte subsets) that differentiate children with MIS-C from acute COVID-19, other infectious non-infectious diseases and control children. Further, we have shown the clear discriminating capability of immune signature that distinguishes MIS-C from other clinical syndromes/conditions using PCA, and hierarchical clustering analysis. After clinical resolution of MIS-C, the above-mentioned observed innate and adaptive immune cellular changes were significantly altered following 6–9 months of recovery. The findings from our study advance our knowledge on the immunology of MIS-C in children and may aid clinicians in differentiating MIS-C from other common tropical conditions. However, further studies focusing on antigen-specific immune responses and mechanistic studies to develop biomarkers of MIS-C and COVID-19 are urgently needed.

## Materials and methods

### Ethics statement

Informed written consent was obtained from parent/guardians of all children along with assent where appropriate. Informed consent was obtained after the nature and possible consequences of the studies were explained. The Internal Ethics Committee (IEC) of the participating institutes approved the study. The study was also registered with Clinical Trials registry (clinicaltrial.gov. No: NCT04844242).

### Study setting and population

Children of either sex, aged 12 months and under 18 years, admitted to the Institute of Child Health, Dr Mehta’s Children Hospital, Rainbow Children’s Hospital, and Kanchi Kamakoti CHILDS Trust Hospital, India from 1 December 2020 to 30 May 2021 with MIS-C, acute COVID-19, other infectious (acute dengue, scrub Typhus and enteric fever) and non-infectious diseases (systemic lupus erythematosus (SLE), diabetic ketoacidosis (DKA), KD) were included in this study. Children who were admitted for elective surgeries were included as controls. For analysis, children were classified into 5 groups: 1) MIS-C (n = 21); 2) acute COVID-19 (n = 23): other infectious diseases (n = 19); 4) non-infectious diseases (n = 21); and 5) healthy controls (n = 14) (children who were SARS-CoV-2 RT-PCR negative and seronegative were in the hospital for elective surgery). Peripheral blood sampling in all children was done prior to receiving any immunomodulatory treatment (pre-IVIG/steroids). Follow-up samples were collected from MIS-C children at 6 to 9 months post-recovery (n = 12). All of the MIS-C children received some kind of immunomodulatory therapy, either with IVIG or steroids. Blood was collected in EDTA tubes (BD Biosciences) and/or heparinized tubes and processed within 4 hours of collection. Study staff involved in immunological assays were blinded to the clinical data. Acute COVID-19 disease and severity of COVID-19 was defined according to the Ministry of Health and Family Welfare (MOHFW) / WHO guidelines [33] and children with MIS-C were diagnosed and treated according to the CDC and WHO definition for MIS-C [34,35]. Dengue fever was confirmed by either serology or NS1 positivity. Scrub typhus was confirmed by serology (IgM) or ELISA. Confirmed culture was used to diagnose Enteric fever. Other conditions like DKA, JIA and GBS were diagnosed as per clinician’s discretion.

### *Ex-vivo* analysis

Leukocyte counts and differentials were performed on all individuals using an AcT5 Diff hematology analyzer (Beckman Coulter). All antibodies used in the study were from BD Biosciences (San Jose, CA), BD Pharmingen (San Diego, CA), eBioscience (San Diego, CA), or R&D Systems (Minneapolis, MN). Briefly, 250ul aliquot of whole blood was added to a cocktail of monoclonal antibodies specific for various immune cell types (S4 Table.). Following 30 min of incubation at room temperature, erythrocytes were lysed using 2 ml of FACS lysing solution (BD Biosciences Pharmingen), and cells were washed twice with 2 ml of PBS and suspended in 200 ul of PBS (Lonza, Walkersville, MD). Naive cells were classified as CD45RA^+^ CCR7^+^ CD95^-^ CD28^+^, central memory cells (T_CM_) as CD45RA^-^ CCR7^+^ CD95^+^ CD28^+^, effector memory cells (T_EM_) as CD45RA^-^CCR7^-^ CD95^+^ CD28, Terminal effector memory cells (T_TEM_) as CD45RA^-^ CCR7^-^ CD95^+^ CD28^-^, stem cell memory (T_SCM_) as CD45RA^+^ CCR7^+^ CD95^+^ CD28^+^ and transitional memory cells (T_TM_) as CD45RA^+^ CCR7^-^ CD95^+^ CD28^+^ [36]. Regulatory T cell were classified as CD4^+^ CD25^+^ Foxp3^+^ CD127dim [36]. Naive B cells were classified as CD45^+^ CD19^+^ CD21^+^ CD27^-^; classical memory B cells as CD45^+^ CD19^+^ CD21^+^ CD27^+^; activated memory B cells as CD45^+^ CD19^+^ CD21^-^ CD27^+^; atypical memory B cells as CD45^+^ CD19^+^ CD21^-^CD27^-^; immature B cells as CD45^+^ CD19^+^ CD21^+^ CD10^+^; and plasma cells as CD45^+^ CD19^+^ CD21^-^ CD20^-^ [37]. Plasmacytoid DCs were classified as (Lin–HLA-DR^+^ CD123^+^) and myeloid DCs (Lin–HLA-DR^+^ CD11c^+^). Classical monocytes were classified as CD45^+^ HLA-DR^+^ CD14^hi^ CD16^-^; intermediate monocytes as CD45+ HLA-DR+ CD14^hi^ CD16^dim^ and non-classical monocytes were classified as CD45+ HLA- DR+CD14^dim^CD16^hi^. Myeloid-derived suppressor cells (MDSCs) were classified as CD45^+^ HLA-DR^+^ CD14^hi^ CD33^+^CD11b^-^. Eight-color flow cytometry was performed on a FACS Canto II flow cytometer with FACS DIVA software, version 6 (Becton Dickinson). The gating was set by forward and side scatter, and 1,00000 gated events were acquired. Data were collected and analyzed using FLOW JO software 10.8.0 (TreeStar, Ashland, OR). Leukocytes were gated using CD45 expression versus side scatter [38,39]. Absolute counts of the subpopulations were calculated based on the equation: Absolute number/mm^3^ of Leukocytes subset = [percent of subset × total number of white blood cells per mm^3^] / 100) [40].

### Statistical analysis

Data were entered into an MS excel sheet and analyzed. Descriptive statistics of variables presented as absolute numbers with percentages and mean (SD) were arrived, as applicable. Geometric means (GM) were used for measurements of central tendency. Statistically significant differences between MIS-C, acute COVID-19, other infectious diseases, non-infectious diseases and controls were analyzed by means of the Kruskal-Wallis test with Dunn’s multiple comparisons. Pre-treatment and post-recovery levels were compared by means of the Wilcoxon signed rank test. Statistical analyses were performed using Graph-Pad PRISM Version 9.0 (GraphPad Software, CA, USA). JMP15 software was used to generate PCA, heat map with dendrogram.

## Supporting information

S1 Fig Gating strategy for T cell subsets. (A). A representative flow cytometry plot showing the gating strategy for estimation of CD4+ T cell memory subsets. Naïve cells (TN) were classified as CD45RA+ CCR7+ CD95- CD28+, central memory cells (TCM) as CD45RA- CCR7+ CD95+ CD28+, effector memory cells (TEM) as CD45RA- CCR7- CD95+ CD28-, Terminal effector memory cells (TE) as CD45RA- CCR7- CD95+ CD28-, stem cell memory (TSCM) as CD45RA+ CCR7+ CD95+ CD28+, transitional memory cells (TTM) as CD45RA+ CCR7- CD95+ CD28+. (B). A representative flow cytometry plot showing the gating strategy for estimation of CD8+ T cell memory subsets. Naïve cells (TN) were classified as CD45RA+ CCR7+ CD95- CD28+, central memory cells (TCM) as CD45RA- CCR7+ CD95+ CD28+, effector memory cells (TEM) as CD45RA- CCR7- CD95+ CD28-, Terminal effector memory cells (TE) as CD45RA- CCR7- CD95+ CD28-, stem cell memory (TSCM) as CD45RA+ CCR7+ CD95+ CD28+, transitional memory cells (TTM) as CD45RA+ CCR7- CD95+ CD28+. (C). A representative flow cytometry plot showing the gating strategy for estimation of Regulatory T cells. Regulatory T cells were defined as CD4+ CD25+ Foxp3+ CD127dim.
(PDF)

S2 Fig Gating strategy for B cell subsets. A representative flow cytometry plot showing the gating strategy for estimation of naïve, immature, classical memory (CM), activated memory (AM), Atypical memory (ATM), immature and plasma cells from CD45^+^ CD19^+^ cells. Naïve cells were classified as CD21^+^ CD27^-^; classical memory (CM) cells as CD21^+^ CD27^+^; activated memory (AM) cells as CD21^-^ CD27^+^; Atypical memory (ATM) cell as CD21^-^ CD27^-^; immature B cells as CD21^+^ CD10^+^; and plasma cells as CD21^-^ CD27^-^.
(PDF)

S3 Fig Gating strategy for myeloid cell subsets. **(A)** An illustrative flow cytometry plot depicting the gating strategy of plasmacytoid (pDC) and myeloid DCs (mDC). Plasmacytoid DC were classified as (Lin–HLA-DR+ CD123+) and myeloid DCs as (Lin–HLA-DR+ CD11c+). **(B)** An illustrative flow cytometry plot depicting the gating strategy of myeloid-derived suppressor cells **(**MDSC). MDSCs were defined by the expression of CD45^+^, CD33^+^, HLA-DR^-^ CD11b^+^. **(C)** A representative flow cytometry plot showing the gating strategy for estimation of monocyte subsets. Classical monocytes were classified as CD45^+^ HLA-DR^+^ CD14^hi^CD16^–^; intermediate monocytes as CD45^+^ HLA-DR^+^ CD14^hi^ CD16^dim^ and non-classical monocytes were classified as CD45^+^HLADR^+^ CD14^dim^CD16^hi^.
(PDF)

S4 Fig Hematology parameters and cellular subsets in MIS-C children post-recovery and control children at baseline. (A) Analysis of WBC, RBC, Hb, HCT and platelets, lymphocytes, monocytes, neutrophils, eosinophils, basophils and neutrophils/lymphocytes ratio were shown for MIS-C children 6–9 months post-recovery [n = 12] and controls [n = 14]. (B) Absolute numbers of CD4^+^ T cell subsets were shown for MIS-C children 6–9 months post-recovery [n = 12] and controls [n = 14]. (C) Absolute numbers of CD8^+^ T cell subsets were shown for MIS-C children 6–9 months post-recovery [n = 12] and controls [n = 14]. (D) Absolute numbers of B cell subsets were shown for MIS-C children 6–9 months post-recovery [n = 12] and controls [n = 14]. (E) Absolute numbers of DC and monocyte cell subsets were shown for MIS-C children 6–9 months post-recovery [n = 12] and controls [n = 14]. The data are represented as scatter violin plots with each circle representing a single individual. p values were calculated using the Mann-Whitney U test.
(PDF)

S1 Table Additional features of COVID-19 children (RT-PCR positive). (DOCX)

S2 Table Additional features of children with other infections. (DOCX)

S3 Table Additional features of children with non-infective aetiology. (DOCX)

S4 Table Antibodies and clones used for *ex-vivo* analysis. (DOC)
