# Supplementary material for: Unique cellular immune signatures of multisystem inflammatory syndrome in children
Source: PLoS Pathog. 2022 Nov 2;18(11):e1010915. doi: 10.1371/journal.ppat.1010915 (PMC9629618; doi:10.1371/journal.ppat.1010915)

S1.Fig

Gating Strategy for CD4$^+$ memory T Cell Subsets

(A)

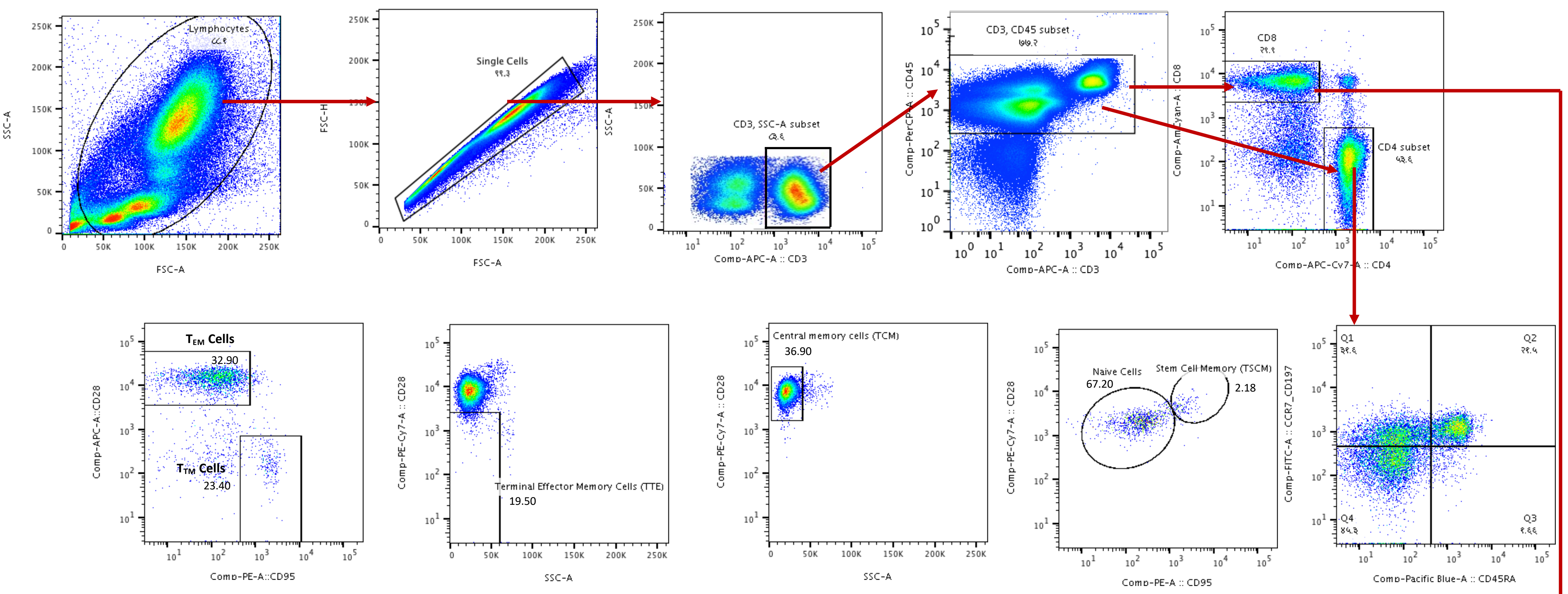

Gating Strategy for CD8$^+$ memory T Cell Subsets

(B)

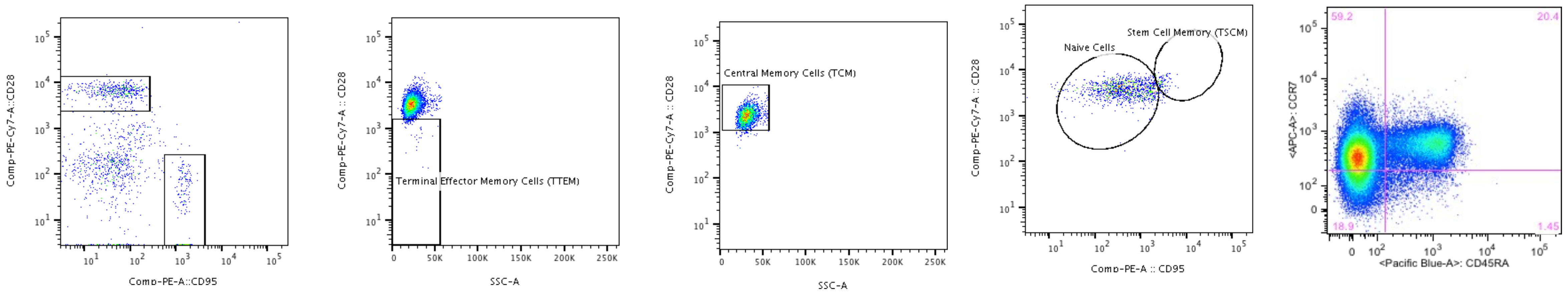

Gating Strategy for Regulatory T Cell Subsets

(C)

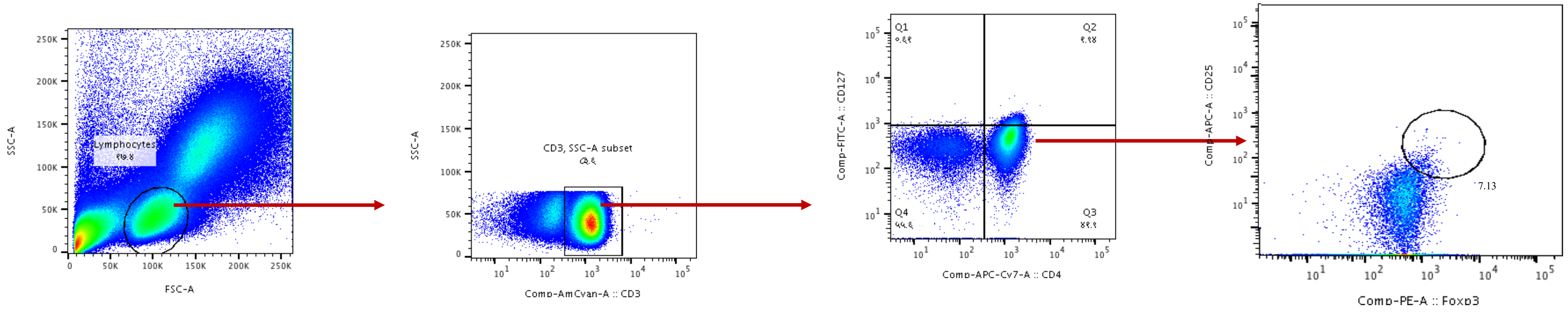

Supplement: S1 Fig — (A). A representative flow cytometry plot showing the gating strategy for estimation of CD4+ T cell memory subsets. Naïve cells (TN) were classified as CD45RA+ CCR7+ CD95- CD28+, central memory cells (TCM) as CD45RA- CCR7+ CD95+ CD28+, effector memory cells (TEM) as CD45RA- CCR7- CD95+ CD28-, Terminal effector memory cells (TE) as CD45RA- CCR7- CD95+ CD28-, stem cell memory (TSCM) as CD45RA+ CCR7+ CD95+ CD28+, transitional memory cells (TTM) as CD45RA+ CCR7- CD95+ CD28+. (B). A representative flow cytometry plot showing the gating strategy for estimation of CD8+ T cell memory subsets. Naïve cells (TN) were classified as CD45RA+ CCR7+ CD95- CD28+, central memory cells (TCM) as CD45RA- CCR7+ CD95+ CD28+, effector memory cells (TEM) as CD45RA- CCR7- CD95+ CD28-, Terminal effector memory cells (TE) as CD45RA- CCR7- CD95+ CD28-, stem cell memory (TSCM) as CD45RA+ CCR7+ CD95+ CD28+, transitional memory cells (TTM) as CD45RA+ CCR7- CD95+ CD28+. (C). A representative flow cytometry plot showing the gating strategy for estimation of Regulatory T cells. Regulatory T cells were defined as CD4+ CD25+ Foxp3+ CD127dim. (PDF) [file ppat.1010915.s001.pdf]
