# Supplementary figures and images for: Unique cellular immune signatures of multisystem inflammatory syndrome in children
Source: PLoS Pathog. 2022 Nov 2;18(11):e1010915. doi: 10.1371/journal.ppat.1010915 (PMC9629618; doi:10.1371/journal.ppat.1010915)

## Gating Strategy for B cell subsets

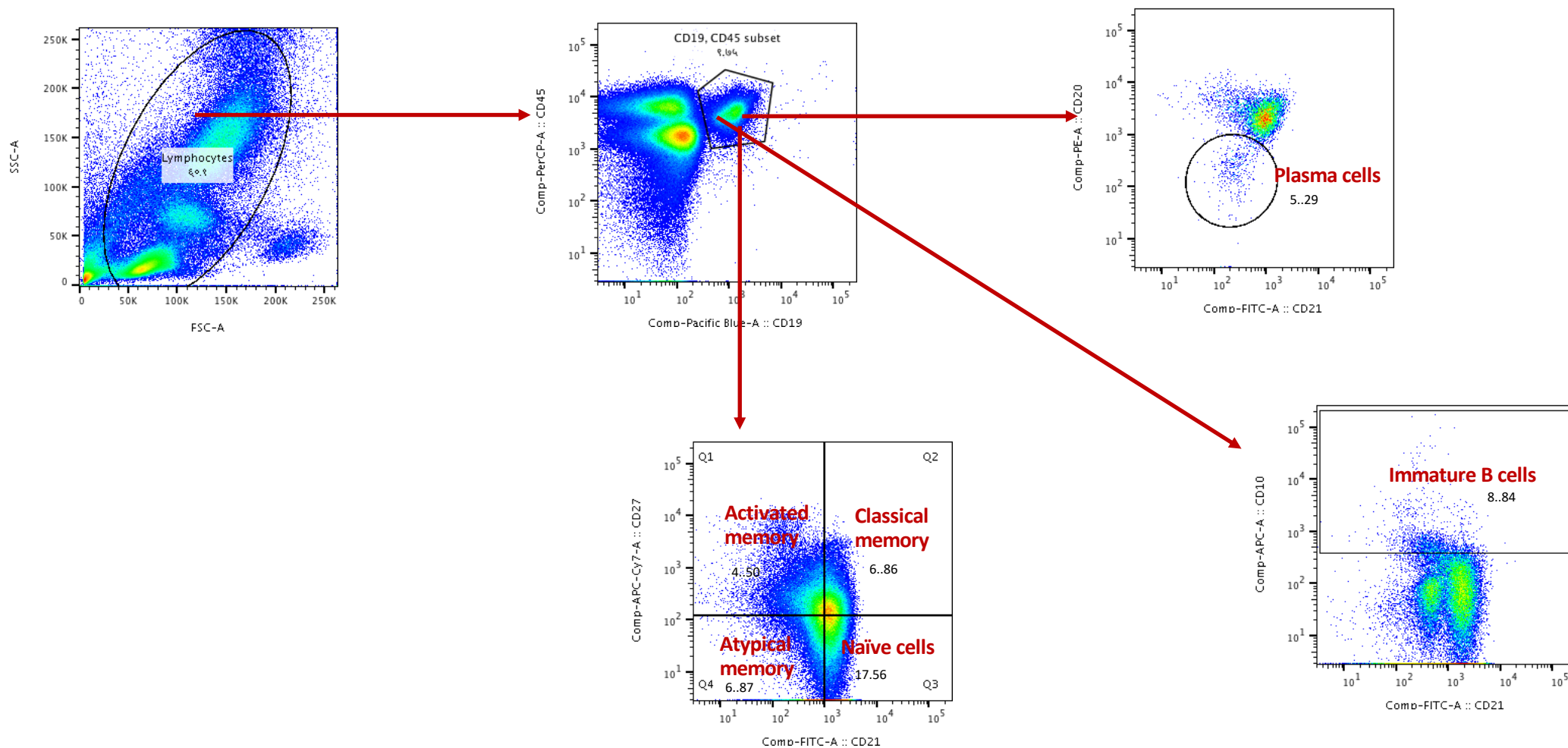

Supplement: S2 Fig — A representative flow cytometry plot showing the gating strategy for estimation of naïve, immature, classical memory (CM), activated memory (AM), Atypical memory (ATM), immature and plasma cells from CD45+ CD19+ cells. Naïve cells were classified as CD21+ CD27-; classical memory (CM) cells as CD21+ CD27+; activated memory (AM) cells as CD21- CD27+; Atypical memory (ATM) cell as CD21- CD27-; immature B cells as CD21+ CD10+; and plasma cells as CD21- CD27-. (PDF) [file ppat.1010915.s002.pdf]

# Gating Strategy for Myeloid cell subsets

S3. Fig.

(A)

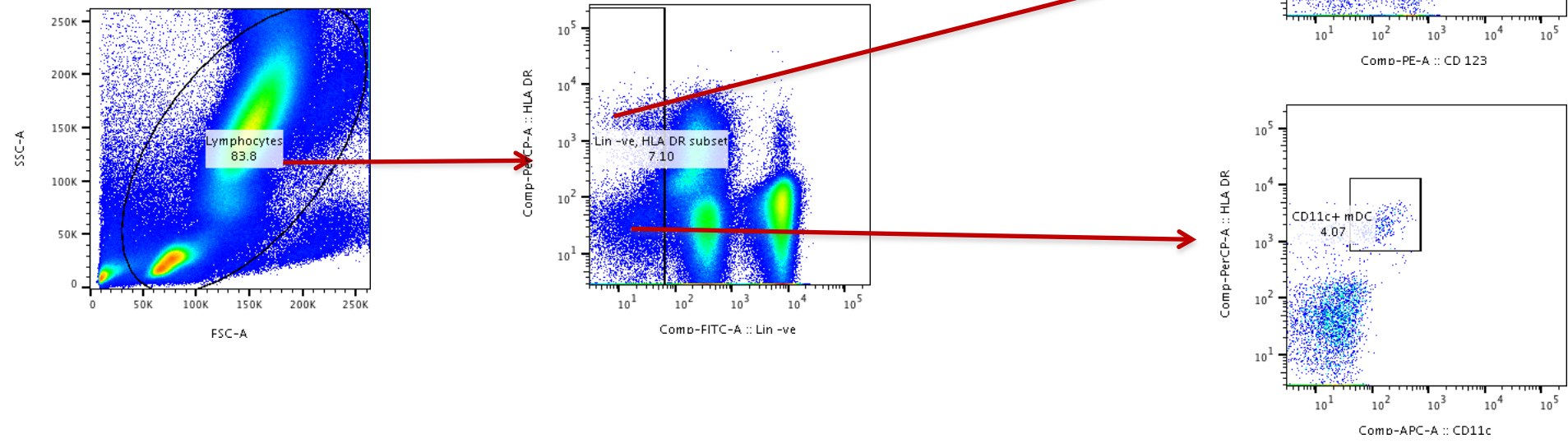

(B)

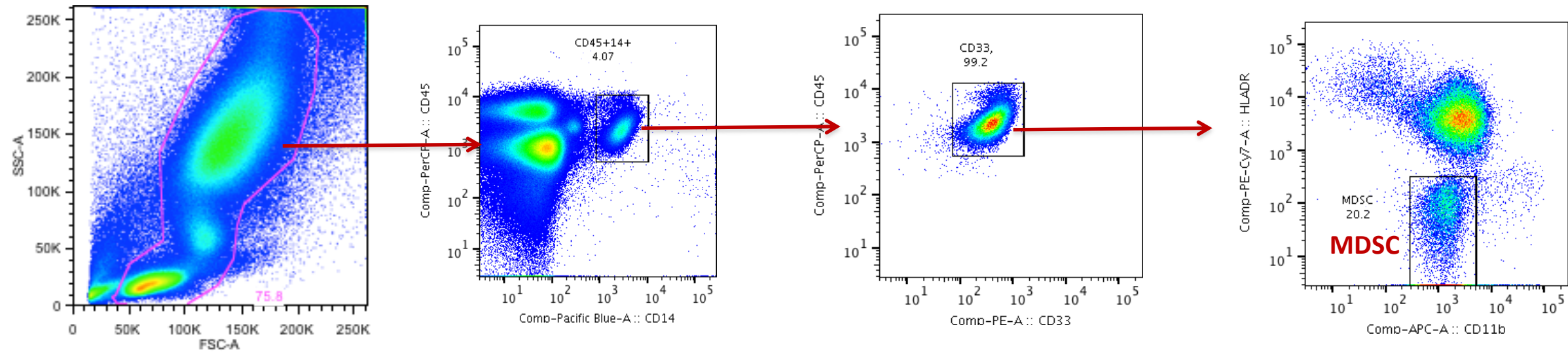

(C)

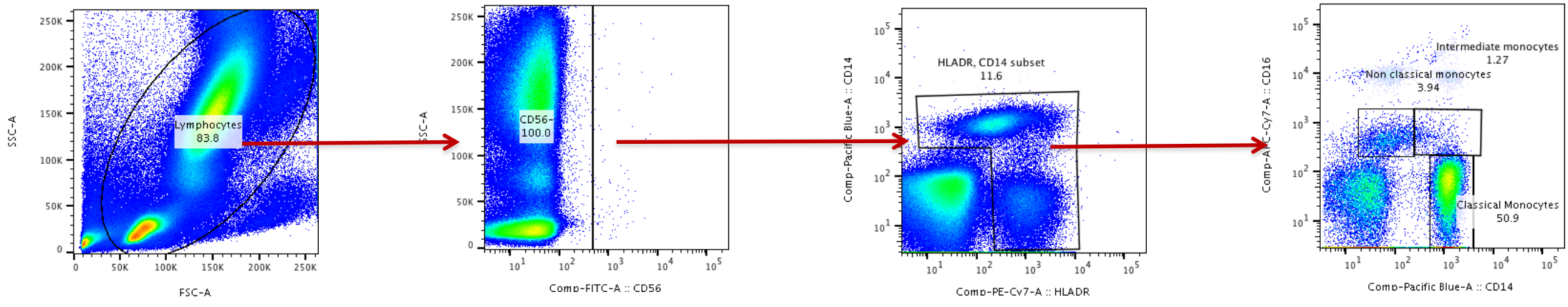

Supplement: S3 Fig — (A) An illustrative flow cytometry plot depicting the gating strategy of plasmacytoid (pDC) and myeloid DCs (mDC). Plasmacytoid DC were classified as (Lin–HLA-DR+ CD123+) and myeloid DCs as (Lin–HLA-DR+ CD11c+). (B) An illustrative flow cytometry plot depicting the gating strategy of myeloid-derived suppressor cells (MDSC). MDSCs were defined by the expression of CD45+, CD33+, HLA-DR- CD11b+. (C) A representative flow cytometry plot showing the gating strategy for estimation of monocyte subsets. Classical monocytes were classified as CD45+ HLA-DR+ CD14hiCD16–; intermediate monocytes as CD45+ HLA-DR+ CD14hi CD16dim and non-classical monocytes were classified as CD45+HLADR+ CD14dimCD16hi. (PDF) [file ppat.1010915.s003.pdf]

S.Fig.4

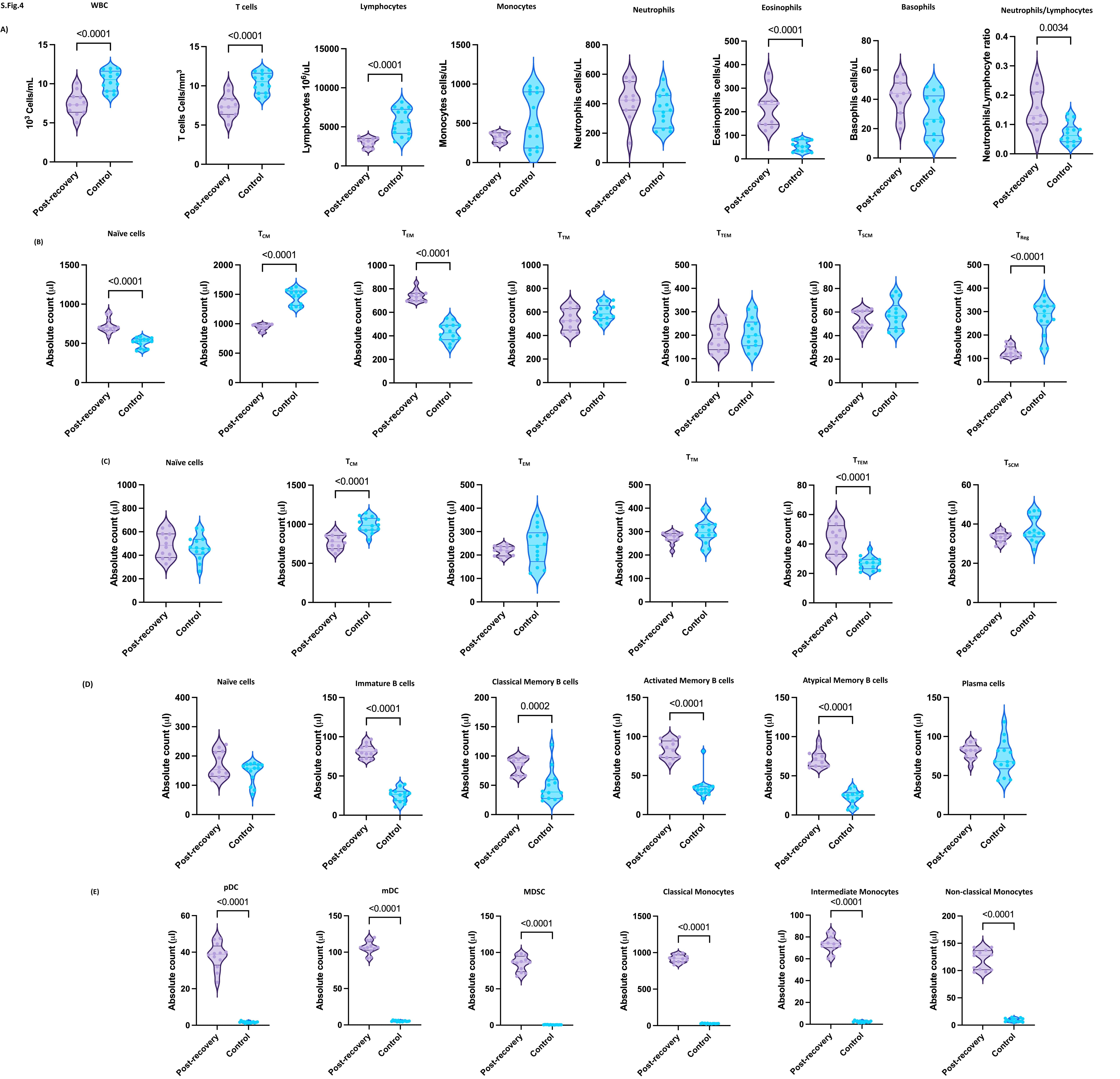

Supplement: S4 Fig — (A) Analysis of WBC, RBC, Hb, HCT and platelets, lymphocytes, monocytes, neutrophils, eosinophils, basophils and neutrophils/lymphocytes ratio were shown for MIS-C children 6–9 months post-recovery [n = 12] and controls [n = 14]. (B) Absolute numbers of CD4+ T cell subsets were shown for MIS-C children 6–9 months post-recovery [n = 12] and controls [n = 14]. (C) Absolute numbers of CD8+ T cell subsets were shown for MIS-C children 6–9 months post-recovery [n = 12] and controls [n = 14]. (D) Absolute numbers of B cell subsets were shown for MIS-C children 6–9 months post-recovery [n = 12] and controls [n = 14]. (E) Absolute numbers of DC and monocyte cell subsets were shown for MIS-C children 6–9 months post-recovery [n = 12] and controls [n = 14]. The data are represented as scatter violin plots with each circle representing a single individual. p values were calculated using the Mann-Whitney U test. (PDF) [file ppat.1010915.s004.pdf]
