# Supplementary material for: Unique cellular immune signatures of multisystem inflammatory syndrome in children
Source: PLoS Pathog. 2022 Nov 2;18(11):e1010915. doi: 10.1371/journal.ppat.1010915 (PMC9629618; doi:10.1371/journal.ppat.1010915)
Supplement: S1 Table — (DOCX) [file ppat.1010915.s005.docx]

**S1.Table: Additional features of COVID-19 children (RT-PCR positive)**

| **Total (n)** | **23** |
| --- | --- |
| **Male n (%)** | 9 (39%) |
| **Age (Median, IQR)** | 6 (1 – 17 yr) |
| **COVID-19 Clinical syndrome**  ***Mild***  ***Moderate***  ***Severe***  ***Asymptomatic*** | **n (%)**  **19 (83%)**  **1 (4%)**  **1 (4%)**  **2 (8%)** |
| **Clinical Symptoms**  ***Fever***  ***Respiratory***  ***Gastrointestinal*** | **19 (83%)**  **5 (22%)**  **12 (52%)** |
| **Underlying conditions (n=4)**  ***Nephrotic syndrome***  ***Dilated Cardiomyopathy***  ***Seizure disorder***  ***ADHD*** | **1**  **1**  **1**  **1** |

**ADHD: Attention Deficit Hyperactivity Disorder**
