# Supplementary material for: Unique cellular immune signatures of multisystem inflammatory syndrome in children
Source: PLoS Pathog. 2022 Nov 2;18(11):e1010915. doi: 10.1371/journal.ppat.1010915 (PMC9629618; doi:10.1371/journal.ppat.1010915)
Supplement: S2 Table — (DOCX) [file ppat.1010915.s006.docx]

**SII. Table: Additional features of children with other infections**

| **Total (n)** | **19** |
| --- | --- |
| **Male n (%)** | 12 (63%) |
| **Age (Median, IQR)** | 4.5 y (1 – 12 y) |
| **Diagnosis**  ***Dengue fever***  ***Scrub typhus***  ***Typhoid***  ***Acinetobacter sepsis***  ***Urinary tract infection***  ***No microorganism isolated*** | **n (%)**  **3 (16%)**  **5 (26%)**  **3 (16%)**  **2 (10%)**  **3 (16%)**  **3 (16%)** |
| **Clinical Symptoms**  ***Fever***  ***Respiratory***  ***Gastrointestinal***  ***Mucocutaneous*** | **10 (53%)**  **5 (26%)**  **8 (42%)**  **2 (10%)** |
| **Underlying conditions (n=1)**  ***Neurodevelopmental delay*** | **1** |
