# Supplementary material for: Unique cellular immune signatures of multisystem inflammatory syndrome in children
Source: PLoS Pathog. 2022 Nov 2;18(11):e1010915. doi: 10.1371/journal.ppat.1010915 (PMC9629618; doi:10.1371/journal.ppat.1010915)
Supplement: S3 Table — (DOCX) [file ppat.1010915.s007.docx]

**SIII. Table: Additional features of children with non-infective aetiology**

| **Total (n)** | **21** |
| --- | --- |
| **Male n (%)** | 10 (48%) |
| **Age (Median, IQR)** | 7 y (2 – 12y) |
| **Underlying Diagnosis**  ***Diabetic ketoacidosis***  ***Kawasaki’s disease***  ***Juvenile idiopathic arthritis***  ***Guillain-Barre syndrome***  ***Systemic lupus erythematosus***  ***Chronic renal failure***  ***Hypothyroidism*** | **n (%)**  **6 (29%)**  **3 (14%)**  **1 (5%)**  **3 (14%)**  **4 (19%)**  **3 (14%)**  **1 (5%)** |
| **Clinical Symptoms**  ***Fever***  ***Respiratory***  ***Gastrointestinal***  ***Mucocutaneous***  ***Neuromuscular (Headache, abnormal gait etc)***  ***Renal*** | **10 (48%)**  **6 (29%)**  **14 (66%)**  **3 (14%)**  **6 (29%)**  **5 (24%)** |
