# Supplementary material for: Unique cellular immune signatures of multisystem inflammatory syndrome in children
Source: PLoS Pathog. 2022 Nov 2;18(11):e1010915. doi: 10.1371/journal.ppat.1010915 (PMC9629618; doi:10.1371/journal.ppat.1010915)
Supplement: S4 Table — (DOC) [file ppat.1010915.s008.doc]

**S IV. Table: Antibodies and clones used for *ex-vivo* analysis**

| **Antibody** | **Flurochrome** | **Clone** | **Company** |
| --- | --- | --- | --- |
| CCR7 | FITC | 3D12 | BD |
| CD95 | PE | DX2 | BD |
| CD45 | Peridinin chlorophyll protein (PerCP) | 2D1 | BD |
| CD28 | APC | CD28.2 | eBioscience |
| CD4 | APC-H7 | RPA-T4 | BD |
| CD3 | Phycoerythrin (PE) Cy7 | SK7 | BD |
| CD45RA | Pacific blue (PB) | HI100 | Biolegend |
| CD8 | AmCyan | SK1 | BD |
| Lin-ve | FITC | SK7, M5E2, 3G8 | BD |
| CD123 | PE | 9F5 | BD |
| HLA-DR | PerCP | L243 | BD |
| CD11c | APC | S-HCL-3 | BD |
| CD56 | FITC | NCAM16.2 | BD |
| CD33 | PE | P67.6 | BD |
| CD11b | APC | ICRF44 | BioLegend |
| HLA-DR | PE-Cy7 | L243 | BD |
| CD16 | APC-Cy7 | 3G8 | BD |
| CD14 | Pacific Blue | M5E2 | BioLegend |
| CD127 | FITC | eBioRDR5 | eBioscience |
| Foxp3 | PE | 236A/E7 | Invitrogen |
| CD25 | APC | 2A3 | BD |
| CD8 | PE-Cy7 | SK1 | BD |
| CD4 | APC-H7 | RPA-T4 | BD |
| CD3 | AmCyan | SK7 | BD |
| CD21 | FITC | B-ly4 | BD |
| CD20 | PE | L27 | BD |
| CD10 | APC | HI10a | BioLegend |
| CD27 | APC-Cy7 | M-T271 | BioLegend |
| CD19 | Pacific Blue | HIB19 | BioLegend |
